# Efficacy of Quadratus Lumborum Block for Postoperative Pain Management in Single-Port Total Laparoscopic Hysterectomy: A Randomized Observer-Blinded Controlled Trial

**DOI:** 10.3390/medicina61040702

**Published:** 2025-04-11

**Authors:** Jihyun Chung, Seunguk Bang, Sangmook Lee, Youngin Lee, Hyun-Jung Shin, Yoonji Park

**Affiliations:** 1 Department of Anesthesiology and Pain Medicine, Daejeon St. Mary’s Hospital, College of Medicine, The Catholic University of Korea, Seoul 03083, Republic of Korea; anesth126@catholic.ac.kr (J.C.); technobear@naver.com (Y.L.); pyj5648@naver.com (Y.P.); 2 Department of Anesthesiology and Pain Medicine, Seoul National University Bundang Hospital, Seongnam 13620, Republic of Korea; hjshin.anesth@gmail.com; 3 Department of Anesthesiology and Pain Medicine, Seoul National University College of Medicine, Seoul 03080, Republic of Korea

**Keywords:** hysterectomy, laparoscopic, nerve block, postoperative pain, quadratus lumborum block, single port

## Abstract

*Background and Objectives*: Quadratus lumborum block (QLB) is a regional anesthesia technique widely utilized in multimodal analgesia strategies for abdominal surgeries. While a few studies have investigated its efficacy in multiport total laparoscopic hysterectomy (TLH), its effectiveness in single-port TLH remains uncertain. This study aimed to evaluate whether QLB reduces opioid consumption and postoperative pain in patients undergoing single-port TLH. *Materials and Methods*: This randomized, controlled, observer-blinded trial included 64 patients undergoing elective single-port TLH. Participants were randomly allocated to either the QLB group (n = 27) or the control group (n = 29). QLB was performed bilaterally under ultrasound guidance after surgery. The primary outcome was cumulative fentanyl consumption within 24 h postoperatively. Secondary outcomes included pain scores at predefined intervals, time to first opioid demand, the incidence of postoperative nausea and vomiting (PONV), and other complications. *Results*: The 24 h cumulative fentanyl consumption, which was the primary outcome, did not differ significantly between the QLB group, 342.8 [220, 651] mcg, and the control group, 470 [191.6, 648.1] mcg (*p* = 0.714). Similarly, cumulative fentanyl consumption at other time points, including 2 h, 4 h, 8 h, 12 h, 32 h, and 48 h, as well as in the PACU, also showed no significant differences between the two groups. Pain scores measured at these time points, along with the time to first bolus on demand, were comparable between groups. However, PONV occurred more frequently in the QLB group than in the control group (25.9% vs. 3.4%, *p* = 0.023). *Conclusions*: QLB did not significantly reduce opioid consumption, time to first opioid demand, or postoperative pain scores in single-port TLH. However, PONV occurred more frequently in the QLB group. These findings suggest that QLB may have limited analgesic benefits in single-port TLH.

## 1. Introduction

Multimodal analgesia has emerged as an effective strategy for postoperative pain management by combining multiple classes of systemic analgesic medications or various modalities [1]. It has significantly advanced in recent years to reduce pain, minimize opioid consumption, prevent chronic pain, and improve the quality of recovery [2,3,4]. Additionally, regional anesthesia techniques, particularly interfascial plane blocks, play a crucial role in abdominopelvic surgeries as part of multimodal analgesia [1,5].

One such interfascial plane block is the quadratus lumborum block (QLB), which has recently gained attention as a promising innovation. Although QLB was initially introduced by Dr. Blanco, the anterior QLB approach has since become the most widely adopted technique [6,7,8]. This approach involves injecting local anesthetics between the quadratus lumborum muscle and the psoas muscle, providing a wide range of sensory blockade and potentially managing visceral pain through sympathetic blockade, although its effectiveness depends on the extent of local anesthetic spread, which is often inconsistent in clinical practice [9,10].

In addition to multimodal analgesia, the importance of procedure-specific postoperative pain management has also been emphasized in recent years [11]. As a result, numerous studies have been conducted to investigate and validate the efficacy of QLB across various surgical indications [12]. For hysterectomy, procedure-specific pain management protocols have been established for both laparotomy and laparoscopic approaches [13]. However, in laparoscopic surgery, the focus has primarily been on multiport laparoscopic hysterectomy [13]. Recently, advances in minimally invasive surgery have led to the increasing adoption of single-port total laparoscopic hysterectomy (TLH), which differs significantly from the multiport approach in terms of surgical techniques, postoperative pain characteristics, complications, and recovery quality. Despite these differences, procedure-specific protocols tailored to single-port TLH remain underexplored, highlighting a critical gap in the current guidelines.

Given two key considerations—the growing importance of QLB as a crucial component of multimodal analgesia and the need for tailored pain management protocols for single-port TLH—this study aims to evaluate whether QLB can reduce opioid consumption within the first 24 h postoperatively and improve postoperative pain management in patients undergoing single-port TLH.

## 2. Materials and Methods

### 2.1. Study Design and Participants

This randomized, controlled, observer-blinded trial was conducted at Daejeon St. Mary’s Hospital, The Catholic University of Korea, Republic of Korea. This study adhered to the principles outlined in the Declaration of Helsinki. Ethical approval (DC16EISI0086) was obtained from the Institutional Review Board of The Catholic University of Korea on 10 January 2017, and the study was registered prospectively with the Clinical Trial Registry of Korea (KCT0002230). The first patient was enrolled on 10 April 2017, and the study was conducted through 9 July 2018. Written informed consent was obtained from all participants prior to enrollment.

Eligible participants included patients aged 19 to 60 years with an American Society of Anesthesiology (ASA) physical status classification of I to III scheduled for elective total laparoscopic hysterectomy (TLH). Exclusion criteria included the presence of significant coagulopathy, infection at the injection site, allergy to local anesthetics, severe cardiopulmonary disease, body mass index (BMI) > 35 kg/m^2^, neuropathies such as diabetic neuropathy, chronic opioid use, refusal of intravenous patient-controlled analgesia (PCA), inability to understand the visual analog scale (VAS) or PCA usage, and patient preference to receive or avoid quadratus lumborum block (QLB).

### 2.2. Randomization

Participants were randomly assigned to either the QLB group (experimental) or the control group using a computer-generated random number table. Randomization information was sealed in opaque, sequentially numbered envelopes, which were opened immediately before the intervention by a team member not involved in block performance or outcome assessment.

### 2.3. Intraoperative Management

Upon arrival in the operating room, standard monitoring was applied. General anesthesia was induced with propofol (1–2 mg/kg), rocuronium (0.6 mg/kg), and a remifentanil bolus (0.5–1 mcg/kg). Anesthesia was maintained with desflurane (4–6 vol%) and a continuous remifentanil infusion (0.05–0.2 mcg/kg/min), targeting a bispectral index between 40 and 60. At the end of surgery, bilateral QLBs were performed in the experimental group. Neuromuscular blockade was reversed using pyridostigmine and glycopyrrolate, and extubation was conducted once the train-of-four ratio exceeded 95%. Patients were then transferred to the recovery room.

### 2.4. Surgical Technique

All surgeries were performed by one of two experienced gynecologic surgeons. A 1.5–2.0 cm vertical transumbilical incision was made, followed by rectus fasciotomy and peritoneal incision. A single-port system (Octoport™, Dalim, Seoul, Republic of Korea) was used, consisting of a retractor and a cap component with multiple channels for laparoscopic instruments and a scope. Carbon dioxide insufflation was applied to create pneumoperitoneum. A 5 mm rigid 0° or 10 mm 30° laparoscope was used as per surgeon preference. Specimens were extracted vaginally via manual morcellation, avoiding trocar-site pain. Vaginal cuff closure was performed laparoscopically using three extracorporeal figure-eight sutures with 0 Polysorb (Syneture, Mansfield, MA, USA).

### 2.5. Block Technique

QLBs were performed under aseptic conditions using real-time ultrasound guidance (WS80A, Samsung Medicine, Seoul, Republic of Korea) by an expert with over five years of experience. In the lateral position, a 2–7 MHz curved ultrasound probe was placed along the midaxillary line to identify the external oblique, internal oblique, and transversus abdominis muscles, along with the transversus abdominis aponeurosis. The probe was moved posteriorly to visualize the quadratus lumborum, psoas major, and erector spinae muscles using the “shamrock sign”. A 22-gauge, 90 mm Tuohy needle (Taechang Inc., Gongju, Republic of Korea) was inserted in a posterior-to-anterior direction. Needle placement was confirmed between the quadratus lumborum muscle and the psoas muscle using an injection of 0.5–1 mL of saline, followed by the administration of 0.375% ropivacaine combined with epinephrine (5 mcg/mL) to minimize the risk of local anesthetic systemic toxicity (Video S1). The same procedure was then performed on the contralateral side.

### 2.6. Postoperative Management

Postoperative analgesia was managed according to our hospital’s Acute Pain Service protocol. Dexamethasone 5 mg was administered prior to the surgical incision, while intravenous fentanyl 1 mcg/kg, ketorolac 30 mg, paracetamol 1 g, and ramosetron 0.3 mg were given before the end of the surgery. In the recovery room, intravenous patient-controlled analgesia (PCA) was initiated using the Accumate1200 device (Woo Young Medical, Daegu, Republic of Korea), delivering fentanyl in patient-demanded boluses of 0.5 mcg/kg with a 7 min lockout interval and no basal infusion. Postoperative oral analgesics included zaltoprofen (80 mg) and celecoxib (200 mg) every 8 h for three days. Rescue analgesia with tramadol (25 mg) was provided if VAS scores exceeded 4 despite PCA.

### 2.7. Outcomes

The primary outcome was cumulative fentanyl consumption within the first 24 h postoperatively, recorded via PCA device logs. Tramadol usage for rescue analgesia was converted into fentanyl equivalents (25 mg tramadol = 25 mcg fentanyl) based on a 1:1000 conversion ratio, which is commonly reported in the literature and supported by multiple randomized controlled trials comparing tramadol and fentanyl [14,15]. Secondary outcomes included VAS scores recorded in the PACU; 2, 4, 8, 12, 24, 32, and 48 h; time to first opioid demand; and the incidence of postoperative nausea and vomiting (PONV). Additionally, baseline postoperative nausea and vomiting (PONV) risk factors were assessed using the Apfel score, which includes female gender, nonsmoker status, history of PONV, and postoperative opioid usage. Complications such as vascular punctures during QLB were also documented.

### 2.8. Statistical Analysis

Based on a pilot study where 24 h fentanyl consumption was 246.3 ± 85.5 mcg, a clinical difference of 30% (73.89 mcg) was deemed significant. Considering a dropout rate of 10%, with an alpha level of 0.05 and power of 90%, we calculated a required sample size of 64 participants (32 per group).

Statistical analyses were conducted using SPSS version 20 (SPSS Inc., Chicago, IL, USA). The normality of the data was assessed using the Kolmogorov–Smirnov test. Continuous variables were compared using the unpaired *t*-test or Mann–Whitney U test, as appropriate, and are presented as mean ± standard deviation (SD) or median [interquartile range (IQR)]. Categorical data were analyzed using the Chi-squared or Fisher’s exact tests. A *p*-value < 0.05 was considered statistically significant.

## 3. Results

**Figure 1:**
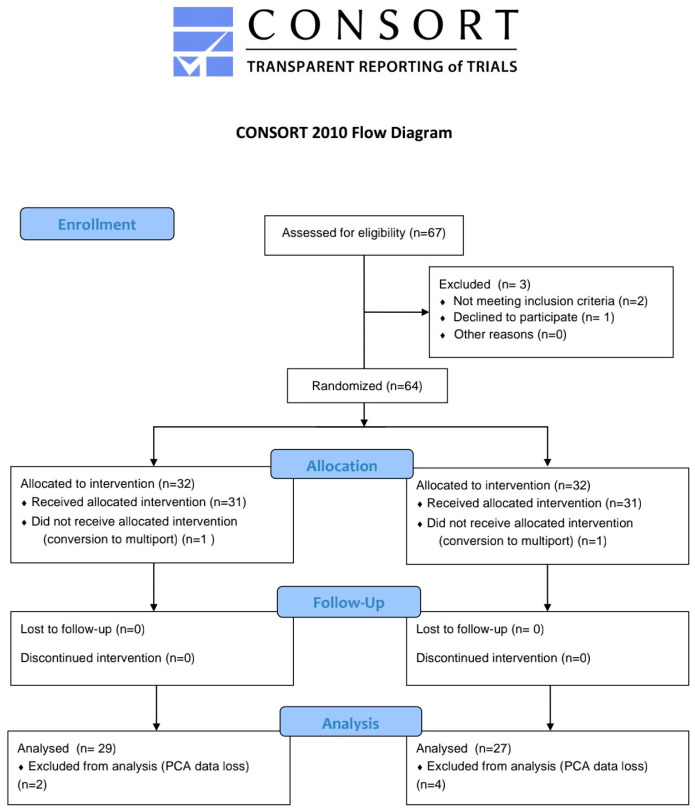
Consort flow diagram.

A total of 64 patients were enrolled in the study, with 32 patients in each group. However, six patients were excluded due to PCA data backup errors, and two patients were excluded after conversion to a three-port approach during surgery. Consequently, the final analysis included 27 patients in the QLB group and 29 patients in the control group (Figure 1). Baseline demographic characteristics are presented in Table 1. Except for height, there were no significant differences between the two groups.

Baseline demographic characteristics are presented in Table 1. Except for height, there were no significant differences between the two groups.

The 24 h cumulative fentanyl consumption was 470 [191.6, 648.1] mcg in the control group and 342.8 [220, 651] mcg in the QLB group, with no statistically significant difference between the groups (*p* = 0.714). Similarly, there were no significant differences in cumulative fentanyl consumption at 2 h, 4 h, 8 h, 12 h, 24 h, 32 h, and 48 h including the PACU (Table 2).

The time to first bolus on demand was comparable between the control group and the QLB group (16 [10–26.5] vs. 14 [10,11,12,13,14,15,16,17,18,19,20,21,22,23,24,25,26], *p* = 0.204). Postoperative VAS scores also showed no statistically significant differences between the two groups (Table 3).

However, there was a significant difference in the incidence of postoperative nausea and vomiting (PONV) between the groups. Despite comparable Apfel risk scores between the groups, PONV occurred in seven patients (25.9%) in the QLB group compared to only one patient (3.4%) in the control group (*p* = 0.023). There were no complications related to the QLB procedure itself. However, one patient in the QLB group experienced mild motor weakness, which was resolved without further issues. No other adverse events were reported.

## 4. Discussion

This study aimed to evaluate the analgesic efficacy of the quadratus lumborum block (QLB) in single-port total laparoscopic hysterectomy (TLH). The primary outcome, cumulative opioid consumption during the first 24 h postoperatively, showed no statistically significant difference between the two groups. Additionally, there were no differences in opioid consumption, pain scores, or time to first bolus administration assessed from the recovery room up to 48 h postoperatively.

Among the various approaches to QLB, anterior QLB typically provides a block of the ventral rami of the T8/9-L1/3 intercostal nerves. It also has the potential to control visceral pain due to the spread of local anesthetic into the thoracic paravertebral space through the lateral arcuate ligament [10,16,17]. Despite this theoretical advantage, our study did not find a significant difference in analgesic outcomes between the QLB and control groups for single-port TLH.

Several studies support findings similar to ours. Hansen et al. [18] investigated the analgesic efficacy of trocar site infiltration versus transmuscular QLB (TQLB) in 70 patients undergoing TLH with a four-port technique. They found no significant difference in the primary outcome, 12 h opioid consumption (morphine-equivalent dose, 58.4 vs. 62.9 mg), or in other time intervals, pain scores, or time to first opioid administration. Similarly, She et al. [19] compared QLB to placebo in 92 patients undergoing three-port TLH and reported no significant difference in cumulative sufentanil consumption (QLB: 0.08 [0.00 to 0.21] vs. placebo: 0.12 [0.03 to 0.23], *p* = 0.268).

QLB is known to provide effective analgesia for somatic pain and is also hypothesized to reduce visceral pain by facilitating the spread of local anesthetic into the TPVS and blocking the sympathetic chain. However, several factors may explain why QLB did not significantly reduce opioid consumption compared to the control group in this study.

First, TLH tends to induce more intense visceral pain than somatic pain, making the management of visceral pain a critical component for reducing opioid consumption. Choi et al. [20] reported that in a study of 50 patients undergoing TLH, visceral pain consistently exceeded somatic pain in intensity during the first 72 h postoperatively.

Second, nociceptive pathways contributing to pain after TLH extend beyond the established visceral analgesic range of QLB (T8–L1). Pelvic visceral pain primarily originates from pelvic organs such as the uterus and vagina. These structures are innervated by both sympathetic and parasympathetic pathways. The sympathetic fibers arise from the T10 to L2 spinal cord segments via the lumbar splanchnic nerves, while the parasympathetic fibers originate from the S2 to S4 segments and travel via the pelvic splanchnic nerves. Both pathways ultimately contribute to the formation of the superior and inferior hypogastric plexuses [21,22,23,24,25].

Nociceptive signals from the uterus and upper vagina are mainly transmitted via the hypogastric plexus to the thoracolumbar spinal segments (T10–L1), whereas signals from the lower vagina are transmitted via the pelvic splanchnic nerves to the sacral spinal cord (S2–S4) [25]. The cervix acts as a watershed region, with afferent fibers projecting through both pathways. Importantly, the sacral component of visceral pain, particularly that resulting from vaginal incisions and disruptions, falls outside the typical sensory coverage of QLB.

Additionally, one of the most common referred pains after laparoscopic surgery is shoulder pain, which is caused by irritation of the central tendon of the diaphragm. The phrenic nerve, originating from C3–C5 spinal segments, provides sensory innervation to the diaphragm and transmits afferent signals to the cervical spinal cord, resulting in referred pain to the shoulder and trapezius regions [26]. This phrenic-nerve-mediated referred pain is known to be severe and clinically significant. One study investigating postoperative pain after TLH found that approximately 90% of patients reported shoulder pain within the first three postoperative days, and nearly two-thirds of patients who required analgesics did so for shoulder pain [20]. As this referred pain originates from the phrenic nerve (C3–C5), it is also outside the coverage area of QLB.

Third, the proposed mechanism of visceral analgesia by QLB, which relies on the spread of local anesthetics to the TPVS and subsequent sympathetic blockade, remains controversial due to its variability. Several anatomical and imaging studies have shown that TPVS spread does not consistently occur after QLB, which could further limit its ability to effectively block visceral pain. Flynn et al. [27] performed thoracic paravertebral blocks in 10 specimens using 30 mL of methylene blue dye, demonstrating successful TPVS spread in 9 cases. However, they reported no dye spread into the space anterior to the quadratus lumborum muscle or around the psoas muscle. Similarly, Gadson et al. [28] used contrast imaging in fresh cadavers, performing TPVB on one side and TQLB on the other, and found no spread from the TQL space to the TPVS. These findings question the proposed mechanism of QLB for reducing visceral pain, which assumes that the QL space and TPVS are connected via the lateral arcuate ligament of the diaphragm.

Finally, somatic pain after TLH primarily arises from port-site skin incisions, disruption and stretching of the muscle fascia during port placement, and irritation of the parietal peritoneum. In single-port TLH, port-site-related pain is inherently less severe compared to multiport procedures due to the reduced number of incisions. Moreover, while parietal peritoneal pain was traditionally considered purely somatic, recent studies have demonstrated that the parietal peritoneum has dual innervation, receiving input from both somatic (lower intercostal and upper lumbar nerves) and visceral (vagal and spinal afferent) fibers [25]. As a result, the somatic pain that QLB is capable of alleviating may be confined to specific components such as skin incisions and muscle fascia disruption and stretching, without fully addressing other port-site-related pain mechanisms. Given this, QLB may demonstrate greater efficacy in surgical settings where somatic pain constitutes a larger proportion of postoperative discomfort. For example, QLB may provide greater analgesic benefits in surgeries such as ovarian cystectomy or myomectomy performed via multiport laparoscopy, where visceral pain is less significant but somatic pain from multiple port-site incisions and muscle fascia disruption is more pronounced compared to single-port procedures. Previous studies have reported that QLB provided effective analgesia in such multiport laparoscopic gynecologic surgeries [29,30]. Similarly, in open surgeries such as total abdominal hysterectomy, where the somatic component of postoperative pain is relatively stronger than in TLH, QLB has been shown to reduce opioid consumption [31,32,33]. These findings suggest that the analgesic efficacy of QLB may depend on the relative contribution of somatic pain in the specific surgical setting.

Taken together, these factors may account for the lack of a significant difference in opioid consumption between the QLB and control groups in this study.

Nevertheless, while our findings align with these limitations, other studies have shown an opioid-sparing effect of QLB in TLH. For example, one study found that TQLB reduced 24 h cumulative morphine consumption compared to subcostal TAP blocks [34]. Another study by Jadon et al. [35] reported reduced 24 h fentanyl consumption (167 vs. 226 mcg, *p* < 0.0001) in the TQLB group compared to a sham block group. These results, however, may reflect the limited effectiveness of subcostal TAP blocks in addressing somatic pain from lower abdominal port incisions, while TQLB could partially address this somatic pain [5,36]. In contrast, our study focused on single-port TLH, where somatic pain is already minimized compared to multiport techniques. With minimal somatic pain and predominantly visceral pain, the impact of QLB on overall pain and opioid consumption appears negligible.

Furthermore, in our study, despite identical prophylactic antiemetic regimens administered to both groups (dexamethasone 5 mg before surgical incision and ramosetron before the end of surgery), the QLB group exhibited a higher incidence of PONV. This difference was unlikely due to baseline risk variations, as evidenced by comparable Apfel scores between the groups. Additionally, since QLB procedures were performed postoperatively without observed hypotension, systemic absorption of local anesthetics potentially affecting the central nervous system might have contributed to the increased incidence of PONV. Future pharmacokinetic analyses of local anesthetic plasma levels could further elucidate this mechanism.

Our study has several limitations. First, we did not differentiate between somatic and visceral pain when assessing postoperative pain, nor did we separately evaluate specific visceral pain characteristics, such as CO_2_-induced referred pain to the scapular area or pelvic organ-related pain. A more detailed and specific pain assessment using precise tools like pain mapping or the Short-Form McGill Pain Questionnaire might have provided more comprehensive data and a clearer interpretation of our findings. Furthermore, imaging techniques, such as dye studies, contrast imaging, or magnetic resonance imaging (MRI), were not performed to confirm the accurate distribution of the local anesthetic into the thoracic paravertebral space (TPVS). Such methods could have clarified the analgesic effects of quadratus lumborum block (QLB). Additionally, although we initially calculated an appropriate sample size based on a clinically significant difference of 30% (73.89 mcg), with an alpha of 0.05 and power of 90%, resulting in 32 participants per group, a post hoc analysis indicated that we achieved sufficient statistical power (92.5%). Therefore, our inability to detect a statistically significant difference between the groups suggests that the actual analgesic effect of QLB might be smaller than the 30% reduction we initially anticipated. Given the complexities of performing QLB and potential associated complications, if the analgesic effect indeed falls below this clinically significant threshold, the clinical relevance of routinely employing QLB as part of multimodal analgesia may be limited. Moreover, despite standardized protocols, individual patient variability—such as differences in pain perception, opioid sensitivity, and response to regional anesthesia—may have influenced opioid consumption and pain scores, introducing potential confounding factors. Although both groups followed a uniform multimodal analgesia protocol and surgeries were performed by the same team, these intrinsic patient-specific factors could not be entirely eliminated. Additionally, although all QLB procedures were performed under real-time ultrasound guidance to ensure precise needle placement, we recognize that technical variability, including block placement, injectate dispersion, and volume, could have influenced our results. Future studies with larger sample sizes, more precise pain assessment tools, advanced imaging techniques, chronic pain evaluation, functional recovery, and further standardization of procedural techniques are warranted to better elucidate the true clinical value and effectiveness of QLB. In addition, alternative regional anesthesia techniques, such as the erector spinae plane block (ESPB), paravertebral block (PVB), and splanchnic nerve block, should be explored as potential strategies to improve analgesic efficacy, particularly in managing visceral pain in gynecologic surgery.

Despite its limitations, our study offers significant strengths. While procedure-specific protocols for multiport TLH are well-established, evidence for single-port TLH remains scarce. As single-port TLH becomes increasingly prevalent, our study represents one of the few prospective randomized trials addressing QLB in this context, providing valuable evidence for PROSPECT guidelines.

## 5. Conclusions

Bilateral TQLB did not significantly reduce opioid consumption or pain scores in patients undergoing single-port TLH. While TQLB may have limited benefits in this specific surgical setting, it may still be valuable in other procedures or for managing certain types of somatic pain. Further studies focusing on pain subtypes and optimizing regional block techniques are warranted to clarify its role in multimodal analgesia.

## Figures and Tables

**Table 1:** Demographic data.

	Control (n = 29)	QLB (n = 27)
Age (yr)	47.5 ± 4.6	48.5 ± 8.7
Height (cm)	158.1 ± 4.2	161.7 ± 5.4
Weight (kg)	62.2 ± 8.8	64.0 ± 9.8
BMI (kg/m^2^)	24.3 [22.3, 27.5]	24.4 [22.0, 26.8]
Operation time (min)	145 [112.5, 199]	161 [140, 184]

Continuous variables are presented as mean ± SD; non-normally distributed data are presented as median [IQR]. BMI: body mass index, QLB: quadratus lumborum block.

**Table 2:** Opioid consumption.

	Control Group (n = 29)	QLB Group (n = 27)	Mean Difference (95% CI)	*p*-Value
Interval opioid consumption (mcg)				
0 h–PACU	55.5 [32, 79.1]	33.7 [25, 74.5]	21.3 (−15.1, 25.8)	0.579
PACU–2 h	58.3 [31.8, 86.4]	71.5 [27.5, 133.6]	−13.2 (−48.8, 10.4)	0.206
2–4 h	34 [0, 64.9]	32.6 [0, 79.2]	1.4 (−22.4, 15.4)	0.686
4–8 h	62.7 [0, 100.1]	33.7 [0, 97.3]	29 (−21.8, 53.3)	0.462
8–12 h	29.2 [0, 128.3]	32.6 [0, 79.2]	−3.4 (−23.1, 60.2)	0.410
12–18 h	54.6 [0, 154.2]	64 [0, 158.4]	−9.4 (−61.2, 45.8)	0.841
18–24 h	50.2 [0, 86.4]	28.9 [0, 63.1]	21.3 (−30.8, 87.9)	0.373
24–32 h	65.6 [0, 163.3]	0 [0, 106.2]	65.6 (−3.51, 87.5)	0.104
32–48 h	0 [0, 25.3]	0 [0, 30.2]	0 (−9.6, 20.2)	0.517
Cumulative opioid consumption (mcg)				
PACU	55.5 [32, 79.1]	33.7 [25, 74.5]	21.3 (−15.1, 25.8)	0.579
2 h	108 [69.4, 163.6]	107.2 [40.5, 196.6]	0.8 (−54.8, 27)	0.529
4 h	144.4 [109.4, 228.1]	153.5 [106.1, 252.4]	−9.1 (−67.6, 24.8)	0.406
8 h	208.5 [120, 315.9]	239.9 [106.1, 377.6]	−31.4 (−76.2, 66.7)	0.850
12 h	305.5 [145, 412]	239.9 [128, 441.2]	65.6 (−85.5, 110.3)	0.836
18 h	404.5 [163.5, 508.2]	317.8 [195.3, 475.4]	86.7 (−135.5, 143.5)	0.911
24 h	470 [191.6, 648.1]	342.8 [220, 651]	127.2 (−151.8, 204.1)	0.714
32 h	548.7 [200.4, 878.9]	396.7 [228.5, 712.8]	152 (−126.7, 267.1)	0.456
48 h	548.7 [200.4, 961.8]	424.1 [228.5, 712.8]	124.6 (−128.6, 297.3)	0.441

Continuous variables are presented as mean ± SD; non-normally distributed data are presented as median [IQR]. CI: confidential interval, QLB: quadratus lumborum block, PACU: post-anesthesia care unit.

**Table 3:** Pain score.

	Control Group (n = 29)	QLB Group (n = 27)	*p*-Value
Pain score (VAS)			
PACU	3 [2, 3]	2 [2, 3]	0.395
2 h	2 [2, 3]	2 [2, 3]	0.919
4 h	2 [2, 3]	2 [2, 3]	0.723
8 h	2 [2, 2]	2 [2, 3]	0.772
12 h	2 [2, 3]	2 [2, 2]	0.623
18 h	2 [2, 3]	2 [2, 3]	0.538
24 h	2 [2, 2]	2 [2, 3]	0.755
32 h	2 [2, 3]	2 [2, 2]	0.529
48 h	2 [2, 2]	2 [2, 2]	0.791
Time to first bolus (min)	16 [10, 26.5]	14 [10, 26]	0.204

Non-normally distributed data are presented as median [IQR]. QLB: quadratus lumborum block, PACU: post-anesthesia care unit, VAS: visual analogue scale.

## Data Availability

The datasets used and/or analyzed during the current study are available from the corresponding author on reasonable request.

## Supplementary Materials

The following supporting information can be downloaded at: https://www.mdpi.com/article/10.3390/medicina61040702/s1, Video S1: Ultrasound-Guided Transmuscular Quadratus Lumborum Block (TQLB): Video S1 demonstrates the TQLB procedure, showing the needle approach and the spread of local anesthetic between the psoas major and quadratus lumborum muscles.
